# Familial hypercholesterolemia in very young myocardial infarction

**DOI:** 10.1038/s41598-018-27248-w

**Published:** 2018-06-11

**Authors:** Sha Li, Hui-Wen Zhang, Yuan-Lin Guo, Na-Qiong Wu, Cheng-Gang Zhu, Xi Zhao, Di Sun, Xiong-Yi Gao, Ying Gao, Yan Zhang, Ping Qing, Xiao-Lin Li, Jing Sun, Geng Liu, Qian Dong, Rui-Xia Xu, Chuan-Jue Cui, Jian-Jun Li

**Affiliations:** Department of Cardiology, State Key Laboratory of Cardiovascular Disease, Fu Wai Hospital, National Center for Cardiovascular Diseases, Chinese Academy of Medical Sciences, Peking Union Medical College, BeiLiShi Road 167, Beijing, 100037 China

## Abstract

Familial hypercholesterolemia (FH) is one of the most common causes of premature myocardial infarction (MI). However, The patterns of FH remained unrecognized in clinical care, especially in very young patients (VYPs, ≤35 years) with MI. The present study enrolled a total of 1,093 VYPs (≤35 years) presenting a first MI. Clinical diagnosis of FH was made using Dutch Lipid Clinic Network criteria. Coronary severity was assessed by Gensini score (GS). Patients were followed for a median of 40-months with cardiac death, stroke, MI, post-discharge revascularization or unstable angina as primary endpoints. The detected rates of definite/probable FH were 6.5%. The prevalence reached up to 10.3% in patients ≤25 years. The FH had similar levels of comorbidities but was younger, more likely to be very high risk (VHR) and had higher GS (p < 0.05) than unlikely FH. Notably, the FH on prior lipid-lowering medication presented a lower GS compared to those untreated. Differences in event rates were similar in FH as unlikely FH (11.8% vs. 8.1%, adjusted hazard ratio 1.35 [0.64–2.86], p = 0.434) but patients on treatment improved outcome (6.5% vs. 10.5%, adjusted hazard ratio 0.35[0.13–0.95], p = 0.039). The early identification and treatment might be critical to reduce cardiovascular risk in VYPs with MI.

## Introduction

Familial hypercholesterolemia (FH) is one of the most common serious genetic disorders of cholesterol metabolism, significantly elevating the levels of low-density lipoprotein cholesterol (LDL-C) and risk of premature atherosclerotic disease including myocardial infarction (MI)^1,2^. If heterozygous FH (HeFH) patients are left untreated, it is estimated that 50% of men at the age of 50 years and 30% of women at the age of 60 years will have had an MI^3^. The untreated homozygotes (HoFH) developing clinically significant in early childhood and generally not surviving beyond 30 years^4^.

Recently, the reported prevalence of FH is increasing. In general population, the prevalence of clinical FH was previously considered to be 0.2% (1:500)^5^ but currently estimated to be as high as 0.5% (1:200)^6^. However, it is increasingly recognized that FH remains widely underdiagnosed and undertreated and consequently there are a large proportion of the affected patients identified until a first asymptomatic atherosclerotic disease even the first MI^7^. Unfortunately, data regarding FH among very young patients (VYPs) presenting a first MI is sparse. It is worthy of noting that fewer population surveys have reported FH frequencies in China, the most populous country with a large number of affected individuals and a severe heavy burden of atherosclerotic disease.

In traditional notion, MI in VYPs (≤35 years) represents a very rare disease with an extremely adverse prognosis^8,9^. Perhaps this is one of the reasons for that studies with large sample size in VYP with MI is largely lacking. Most studies were observed in middle-aged or older individuals previously. In China, especially during the past 2 decades, the society and economy accompanied with a rapid pace of life and an extensively unhealthy lifestyle were developed. Thus, an increasing number of the younger people have heart attacks. The limited literature on premature coronary artery disease (CAD) suggested the alarming condition of the VYP with MI particularly in young men and women with FH^7,10,11^. This perception, however, is in discordance with several reports. For example, Akosah KO *et al*.^12,13^ have suggested a high rate of young adults with diagnosed CAD without high LDL-C levels. Based on these considerations, the present study was designed to define very young age as 35 and younger.

In real clinical practice, diagnosis of the FH using clinical criteria is more common and effective. Although genetic screening is a good approach with efficient identification and future personalized medicine or family screening might benefit from knowledge of genomic background, identification of a causative gene variant is not essential for either diagnosis or treatment decisions^14^. As suggested by investigators including Stein and Fazio^15^, the screening focused on subjects with the phenotype is fundamental to facilitate early identification and treatment of FH. The Dutch Lipid Clinic Network (DLCN) criteria is a nationwide screening program for clinical FH. It produces scores that lead to classification of either definite or probable FH and is critical to distinguish patients from conditions with phenotypical presentations to ensure appropriate clinical care and reduce CAD risk later in life.

In an effort to better characterize the VYPs with MI and to delineate the clinical FH, we recruited a large sample size (n = 1093) of patients with MI (≤35 years) from Fuwai Hospital, the largest medical center for cardiovascular diseases in China [more than 10,000 percutaneous coronary interventions (PCIs) annually] taking full advantage of “big data” to (1) identify the patients with FH phenotype, (2) examine the clinical profile of FH and (3) investigate cardiovascular outcomes after the first MI in this population.

## Materials and Methods

### Study population

Our study complied with the Declaration of Helsinki and was approved by the hospital’s ethical review board (Fuwai Hospital & National Center for Cardiovascular Diseases, Beijing, China). Informed written consent was obtained from all patients enrolled in this study at admission to hospital.

The study patients were consecutively enrolled between May 2009 and April 2017 in Fuwai Hospital with coronary angiography (CAG) for their first MI being the screening process. The age criteria for the VYPs were defined as ≤35 years. MI was defined based on two of the following conditions: angina; electrocardiograph changes or elevated enzyme levels. Baseline demographics, cardiovascular risk factors, lipid-lowering treatment (more than 3 months prior to the admission)^16^, and family history were self-reported and collected retrospectively by a trained nurse from the medical records or by direct interview of the patients. Plasma cholesterol was measured within 24 hours of admission to the hospital. Patients with significant hematologic disorders, infectious or systematic inflammatory disease, thyroid dysfunction, severe liver and/or renal insufficiency and malignant disease were excluded.

### Diagnostic criteria for FH

The clinical FH was diagnosed using the DLCN criteria^11^. For the diagnostic classification, individuals on lipid-lowering medications had their untreated LDL-C levels conservatively adjusted by a correction factor that depends on the dose and potency of statins^17^. Specially, the following numerical score definition of FH according to the DLCN was employed: (i) family history of a first-degree relative with known premature CAD or vascular disease (≤55 years for men; ≤60 years for women, 1 point) and/or a first-degree relative with known hypercholesterolemia (1 point) or xanthomas (2 points) or offspring(s) with known hypercholesterolemia (2 point); (ii) personal history of premature CAD (ages as above, 2 points) or cerebral/peripheral vascular disease (ages as above, 1 point) or xanthomas (6 points); untreated LDL-C > 8.5 mmol/L (8 points), 6.5–8.4 mmol/L (5 points), 5.0–6.4 mmol/L (3 points), or 4.0–4.9 mmol/L (1 point); (iii) corneal arcus and molecular diagnosis (monogenic anomalies) were not available and these missing information was counted as zero in this algorithm. Finally, a diagnosis of definite FH was considered if the total score was greater than 8, probable if the score was 6–8, possible if the score was 3–5, and unlikely if the score was below 3 points.

### Risk categorizations

All FH patients are considered to be at a very high cardiovascular risk, but the risk within FH is variable. Several categorizations of FH patients have been reported in the literature^18–20^. In the present study, we grouped our patients according to the National Lipid Association (NLA) recommendations^20^. Patients were considered at very high risk (VHR), if they meet one of the following criteria: secondary prevention, smoking, diabetes, CAD family history, lipoprotein (a) [Lp(a)] > 50 mg/dL or presence of 2 other major risk factors [man aged older than 30 years, high density lipoprotein cholesterol (HDL-C) < 0.4 mg/L, LDL-C > 6.5 mmol/L, hypertension and tendon xanthoma]. Those belonged to VHR were highlighted the susceptibility to significant cardiovascular burden and strengthened the need for a closer follow-up or management.

Several investigators have suggested that angiographic score data should be routinely included into risk evaluation of cardiovascular patients^21^. In the present study, we evaluated the coronary severity of each patient by the Gensini scoring system^22^. The Gensini score (GS) was computed by assigning a severity score to each coronary stenosis according to the degree of luminal narrowing and its geographic importance. Firstly, reductions in the lumen diameter or roentgenographic appearances of the coronary lesion were evaluated as 1 for 1–25% stenosis, 2 for 26–50% stenosis, 4 for 51–75% stenosis, 8 for 76–90% stenosis, 16 for 91–99% stenosis and 32 for total occlusion. Then, the scores were multiplied by the weight coefficient that represented the importance of the lesion’s position. They were 5 for the left main coronary artery, 2.5 for the proximal left anterior descending or proximal left circumflex artery, 1.5 for the mid-region, 1 for the distal left anterior descending or mid-distal region of the left circumflex artery and 0.5 for small vascular branches.

### Follow-up and outcomes

The patients in our hospital had relatively regular visit because the visit is regarded as a part of routine clinical procedures, thus we had relatively stable events data. The patients were informed the follow-up matters during the initial appointment by trained nurses or cardiologists, who were blinded to the results of the laboratory tests.

Specially, patients were prospectively followed up at 6, 12, 24, 36 months using telephone and/or interview after the initial appointment. The primary outcome was the composite of cardiac death, stroke, MI, post-discharge revascularization [PCI/(coronary artery bypass grafting, CABG)] or unstable angina (UA)^23^. Cardiac death was primarily confirmed by the death from the cardiac causes including sudden cardiac death, congestive heart failure, acute MI, severe arrhythmia, stroke, or other structural/functional cardiac diseases. The definition of stroke was the acute cerebral infarction on the basis of the imaging or typical symptoms. MI was diagnosed by a comprehensive evaluation combining the chest pain or equivalent symptom complex, the diagnostic changes in cardiac enzyme levels, and the electrocardiogram. Post-discharge revascularization was the PCI and/or CABG performing during the follow-up. UA was considered if patients reported the chest pain, which was characterized with the new-onset angina and/or rest symptoms and/or increasing duration/severity compared to the previous stable symptoms, the dynamic electrocardiogram changes, but without abnormal change in cardiac enzyme levels.

### Statistical analysis

The values were expressed as the mean ± SD for the continuous variables and the number (percentage) for the categorical variables. The differences of clinical and biochemical parameters among the diagnostic groupings of FH were analyzed using χ^2^-tests, one-way ANOVA and Mann-Whitney test where appropriate. General linear model was used to study the variables compared between the patients with and without lipid-lowering treatment with adjustments for confounding factors, and to calculate the adjusted mean values, respectively. Odds ratio (OR) and 95% confident interval (95% CI) was calculated with multiple logistic model adjusted by potential confounding factors. Cox regression analysis for event-free survival was performed to investigate the predictive value of FH phenotype for recurrent events. Time-to-event curve was constructed using the Kaplan–Meier method to describe the incidence of events over time, and log-rank test was applied to evaluate difference among FH groups. A p-value < 0.05 was considered statistically significant. The statistical analysis was performed with SPSS version 19.0 software (SPSS Inc., Chicago, IL, USA).

## Results

### Baseline characteristics of VYPs with MI

There were a total of 1093 eligible participants of VYPs with MI in the present study (Table 1). The average age of the patients was 31.6 ± 3.6 years and 96.8% (n = 1058) of them were men. The number of 390 patients (35.7%) was already receiving lipid-lowering treatment prior to the admission. 880 (80.5%) patients had LDL-C levels of ≤3.37 mmol/L, 687 (78.1%) of those patients had LDL-C levels of ≤2.59 mmol/L. There were 99 (10.4%) patients had LDL-C levels considered to be alarmingly high (>4.14 mmol/L) in this very young infarction population.

**Table 1:** Characteristics of participants according to the diagnostic probability of FH phenotype.

	All	Diagnostic probability of FH			
		Definite/probable	Possible	Unlikely	P for trend
Number	1093	71	374	648	
Prevalence,%	100.0%	6.5%	34.2%	59.3%	
Men,% (n)	96.8(1058)	97.2(69)	96.5(361)	96.9(628)	0.927
Age (year)	31.6 ± 3.6	31.0 ± 4.9	32.0 ± 3.3	31.5 ± 3.6	**0**.**045**
BMI (kg/m^2^)	27.26 ± 3.97	26.99 ± 3.35	27.95 ± 3.72	26.79 ± 4.19	0.079
Hypertension,% (n)	38.1 (416)	33.8 (24)	42.5 (159)	36.0 (233)	0.174
Diabetes mellitus,% (n)	17.1 (187)	19.7 (14)	18.2 (68)	16.2 (105)	0.779
Current smokers (%)	68.3 (746)	64.8 (46)	69.0 (258)	68.2 (442)	0.784
Family history of CAD,% (n)	21.7 (237)	39.4 (28)	55.9 (209)	0 (0)	**<0**.**001**
TG (mmol/L)	1.74 (1.24–2.41)	1.96 (1.62–2.53)	1.84 (1.27–2.51)	1.66 (1.20–2.31)	**0**.**002**
TC (mmol/L)	4.17 ± 1.30	6.44 ± 1.46	4.43 ± 1.28	3.77 ± 0.97	**<0**.**001**
HDL-C (mmol/L)	0.91 ± 0.25	0.90 ± 0.20	0.90 ± 0.23	0.91 ± 0.27	**0**.**842**
LDL-C (mmol/L)	2.53 ± 1.16	4.86 ± 1.53	2.76 ± 1.10	2.14 ± 0.73	**<0**.**001**
Untreated LDL-C (mmol/L)	3.17 ± 1.56	7.31 ± 1.55	3.44 ± 1.27	2.57 ± 0.79	**<0**.**001**
ApoAI (g/L)	1.14 ± 0.26	1.09 ± 0.26	1.14 ± 0.26	1.15 ± 0.26	0.201
ApoB(g/L)	0.95 ± 0.33	1.44 ± 0.34	1.03 ± 0.33	0.86 ± 0.26	**<0**.**001**
Lp (a) (mg/L)	136.65 (54.47–318.28)	261.30 (114.38–478.27)	142.78 (61.48–329.31)	122.46 (48.67–280.96)	**<0**.**001**
Glucose (mmol/L)	5.72 ± 2.26	6.23 ± 2.82	5.93 ± 2.25	5.54 ± 2.18	**0**.**004**
HbA1C (%)	6.14 ± 2.06	6.35 ± 1.62	6.15 ± 1.37	6.11 ± 2.41	0.615
Hs-CRP (mg/L)	2.41 (1.10–8.12)	3.99 (1.63–10.04)	2.66 (1.32–8.40)	2.16 (0.96–7.14)	**0**.**006**
Lipid-lowering treatment,% (n)	35.7 (390)	69.0 (49)	38.2 (143)	30.6 (198)	**<0**.**001**
VHR,% (n)	92.5 (1011)	98.6 (70)	95.7 (358)	90.0 (583)	**<0**.**001**
Gensini score	22 (4–48)	34 (16–66)	23 (4–50)	20 (2–46)	**<0**.**001**

Data shown are mean ± SD, median (interquartile) or n (%). The bold values indicated statistical significance. FH, familial hypercholesterolemia; BMI, body mass index; CAD, coronary artery disease; TG, triglyceride; TC, total cholesterol; HDL-C, high-density lipoprotein cholesterol; LDL-C, low-density lipoprotein cholesterol; apo, apolipoprotein; Lp(a), Lipoprotein(a); HbA1C, hemoglobin A1C; Hs-CRP, high sensitively C reactive protein; VHR, very high risk.

### Clinical FH prevalence and features

Of the 1093 patients presenting a very advanced MI, there were 71 patients identified as having definite/probable FH, representing a prevalence of 6.5% in this population. Specially, 14 patients (1.3%) were identified as the definite FH and 57 patients (5.2%) were regarded as the probable FH. The prevalence was lower as age increased (≤25 vs. 26–30 vs. 31–35 years 10.3%, 6.9%, 6.0%, p = 0.377) but not significant. The patients on lipid-lowering treatment prior to the admission were found to have a significant higher prevalence than those untreated (treated vs. untreated 12.6%, 3.1%, p < 0.001).

In the subgroup of patients with definite/probable FH, 57.7% (41) were with an alarming LDL-C level of >4.14 mmol/L while none of them got the optimal level of ≤2.56 mmol/L. Compared to those with definite/probable FH, patients with possible FH and unlikely FH had less alarming LDL-C (15.5% and 0%, respectively) but more optimal level (52.7% and 75.6%, respectively). As shown in Table 1, the FH group was younger, more likely to be treated with lipid-lowering drugs, had higher rates of family history of CAD and VHR or higher GS than the unlikely FH. However, these patients had similar levels of comorbidities, such as hypertension, diabetes, obesity and smoking.

In addition to the increasing LDL-C levels in FH, certain lipid indices including Lp(a) have received growing or re-waking interest^24,25^. Hence, we evaluated the association of Lp(a) and clinical FH in this very young infarction patients. It is reported that the mean cholesterol content of total Lp(a) mass is roughly 30%, which is co-measured in total and LDL-C measurements. Given this consideration, we used the LDL-C adjusted for Lp(a) cholesterol to diagnose FH. In this way, 60 (5.5%) definite/probable FH, 362 (33.1%) possible FH and 671 (61.4%) unlikely FH were identified, suggesting 15.5% [(71–60)/71] of definite/probable FH might be explained by cholesterol from Lp(a) in this population (Supplemental Fig. 1). To further investigate the impact of Lp(a) on determining clinical FH, univariate and multivariate regression analyses were performed (Table 2). The crude and adjusted ORs (95%CIs) of Lp(a) for clinical FH were 1.539(1.224–1.935), 1.599(1.247–2.050), respectively. We also performed the receiver operating characteristic (ROC) analysis and found the values of the area under the ROC curve (AUC) for predicting definite/probable FH were significant by Lp(a) (0.681, 95% CI 0.616–0.747, p < 0.001). The cut-off value was 224.05 mg/L (data not shown in the Table).

**Table 2:** Univariate and multivariate logistic regression analysis to determine the independent predictor of FH.

	Univariate			Multivariate		
	OR	95% CI	P Value	OR	95% CI	P Value
TC(mmol/L)	3.548	2.799–4.497	**<0**.**001**	3.677	2.852–4.740	**<0**.**001**
TG(mmol/L)	1.063	0.908–1.245	0.446	1.07	0.904–1.268	0.43
HDL-C(mmol/L)	0.955	0.363–2.514	0.926	0.994	0.344–2.872	0.991
LDL-C(mmol/L)	4.016	3.104–5.196	**<0**.**001**	4.53	3.324–6.174	**<0**.**001**
Lp(a)(mg/L)	1.539	1.224–1.935	**<0**.**001**	1.599	1.247–2.050	**<0**.**001**
Glucose(mmol/L)	1.073	0.980–1.174	0.126	1.072	0.930–1.236	0.339
HbA1C(%)	1.024	0.934–1.122	0.617	1.007	0.875–1.159	0.923
Hs-CRP(mg/L)	1.028	0.978–1.181	0.274	1.017	0.965–1.073	0.521
Family history of CAD	2.507	1.510–4.161	**0**.**025**	2.442	1.447–4.121	**0**.**001**

Logistic regression analyses were performed. Bold values indicated statistical significance. The covariates included age, sex, BMI, hypertension, diabetes, current smoker, HbA1c, Hs-CRP, family history of premature CAD. TC: total cholesterol; TG: triglyceride; LDL-C: low-density lipoprotein cholesterol; HDL-C: high-density lipoprotein cholesterol; Lp(a): Lipoprotein(a); Hs-CRP, high sensitively C reactive protein; HbA1C, hemoglobin A1C; CAD, coronary artery disease;OR: odds ratio. CI: Confidence interval.

Of note, most (77.1%) MI patients were more than 30 years, whereas 22.7% were from 26–29 years and only 6.2% were less than 25 years but the prevalence of definite/probable FH seemed a lower tendency as age increased (≤25 vs. 26–30 vs. 31–35 years 10.3%, 6.9%, 6.0%, p > 0.05). However, it should be noted that the tendency had no statistical significance and the sample of the study population was relatively small to distinguish the difference.

### Lipid-lowering treatment in FH

It is a plain truth that among Chinese individuals with elevated LDL-C, the proportion of those who were aware, treated or controlled for their condition is much lower than in Western populations and should be a reason for great concern^26^. In the present study, the FH patients were identified until they had the asymptomatic MI. Not surprisingly, those patients were undertreated and none of them got the optimal LDL-C even though 69.0% of definite/probable FH were had a lipid-lowering therapy prior to the admission. Nevertheless, we performed the analysis to investigate if this prior treatment or undertreatment could make a difference in their cardiovascular benefit.

As shown in Table 3, patients who were on prior lipid-lowering treatment had a lower LDL-C level regardless of the FH probability. This was also true after the adjustment for age, sex, body mass index (BMI), hypertension, diabetes, smoking, family history of CAD and high sensitivity C-reactive protein (hs-CRP). Interestingly and importantly, among patients with definite/probable FH, the lipid-lowering medication group presented a significant lower GS but showed no difference in number of VHR-associated factors compared to the non-lipid-lowering medication group. When considering patients with possible FH or unlikely FH, the GS was lower but not significant in lipid-lowering medication group while more VHR-associated factors were observed compared to those without lipid-lowering medication.

**Table 3:** Prior lipid-lowering treatment according to FH status.

	Crude Model			Adjusted Model		
	Lipid-lowering treated	Non-lipid-lowering treated	P-value	Lipid-lowering treated	Non-lipid-lowering treated	P-value
**Definite/probable FH**						
LDL-C (mmol/L)	4.09 ± 0.84	6.58 ± 1.30	**<0**.**001**	3.58 ± 0.13	6.52 ± 0.22	**<0**.**001**
Untreated LDL-C (mmol/L)	7.61 ± 1.59	6.64 ± 1.26	**0**.**014**	6.63 ± 0.20	6.69 ± 0.34	0.906
Lp(a) adjusted LDL-C (mmol/L)	3.83 ± 0.86	6.34 ± 1.39	**<0**.**001**	3.25 ± 0.14	6.16 ± 0.24	**<0**.**001**
Number of VHR-associated factors	4.24 ± 1.22	3.77 ± 1.15	0.129	4.23 ± 0.19	3.59 ± 0.33	0.142
Gensini score	26 (15–50)	68 (34–94)	**<0**.**001**	33.40 ± 6.99	77.66 ± 11.95	**0**.**009**
**Possible FH**						
LDL-C (mmol/L)	2.33 ± 0.64	3.03 ± 1.24	**<0**.**001**	2.24 ± 0.13	3.16 ± 0.11	**<0**.**001**
Untreated LDL-C (mmol/L)	4.03 ± 1.09	3.07 ± 1.24	**<0**.**001**	4.05 ± 0.14	3.29 ± 0.12	**<0**.**001**
Lp(a) adjusted LDL-C (mmol/L)	2.12 ± 0.67	2.85 ± 1.23	**<0**.**001**	2.03 ± 0.13	2.97 ± 0.11	**<0**.**001**
Number of VHR-associated factors	4.50 ± 1.11	3.53 ± 1.31	**<0**.**001**	4.66 ± 0.09	3.56 ± 0.08	**<0**.**001**
Gensini score	20 (5–46)	24 (2–54)	0.459	33.44 ± 6.21	43.90 ± 5.39	0.227
**Unlikely FH**						
LDL-C (mmol/L)	1.72 ± 0.43	2.33 ± 0.76	**<0**.**001**	1.73 ± 0.09	2.31 ± 0.08	**<0**.**001**
Untreated LDL-C(mmol/L)	2.91 ± 0.67	2.41 ± 0.80	**<0**.**001**	2.98 ± 0.11	2.58 ± 0.09	**0**.**006**
Lp(a) adjusted LDL-C (mmol/L)	1.55 ± 0.44	2.19 ± 0.76	**<0**.**001**	1.61 ± 0.09	2.16 ± 0.08	**<0**.**001**
Number of VHR-associated factors	3.79 ± 1.17	2.75 ± 1.20	**<0**.**001**	3.81 ± 0.09	2.65 ± 0.08	**<0**.**001**
Gensini score	20 (6–44)	20 (2–48)	0.581	30.99 ± 4.50	31.34 ± 3.72	0.954

General linear models were used to study the variables compared between the patients with and without lipid-lowering treatment. Adjusted models were with adjustment for age, sex, BMI, hypertension, diabetes, smoking, family history of CAD and hs-CRP. FH, familial hypercholesterolemia; LDL-C, low-density lipoprotein cholesterol; Lp(a), Lipoprotein(a); VHR, very high risk.

### FH and vascular outcomes

During a median of 40 months of follow-up, we obtained the data from 1076 patients [17 patients (1.6%) were lost their follow-up]. Among of them, 98 (9.1%) patients presented with at least 1 events (17 cardiac deaths, 1 nonfatal strokes, 9 MIs, 10 revascularizations and 61 UAs). The rate of recurrent events was higher in FH phenotype but not significant (definite/probable vs. possible vs. unlikely FH 11.8%, 10.5%, 8.1%, p = 0.101).

As shown in Table 4. the difference in the events risk of FH status were not statistically significant. The event free times in definite/probable, possible and unlikely FH were 85.8 ± 4.2, 87.8 ± 1.7 and 89.0 ± 1.3 months (Supplemental Fig. 2, p = 0.731). However, we found a significant lower rate of events in lipid-lowering receivers (receivers vs. non-receivers 6.5%, 10.5%, p = 0.017). Additionally, GS and the prior lipid-lowering treatment were associated independently with the events risk (p = 0.039).

**Table 4:** Unadjusted and adjusted Cox proportional HRs for cardiovascular events.

	Crude HR(95%CI)	P value	Adjusted HR(95%CI)	P value
Definite/probable FH vs. unlikely FH	1.30 (0.62–2.73)	0.496	1.35 (0.64–2.86)	0.434
Possible FH vs. unlikely FH	1.13 (0.74–1.71)	0.579	1.11 (0.73–1.69)	0.627
LDL-C	1.07 (0.91–1.25)	0.426	0.78 (0.50–1.21)	0.264
Untreated LDL-C (mmol/L)	1.00 (0.88–1.14)	0.951	1.00 (0.87–1.14)	0.954
Lp (a) adjusted LDL-C (mmol/L)	1.05 (0.89–1.23)	0.572	0.79 (0.51–1.22)	0.288
VHR	1.88 (0.69–5.11)	0.218	1.97 (0.86–6.34)	0.147
Gensini score	1.006 (1.000–1.011)	**0**.**043**	1.013 (1.002–1.024)	**0**.**019**
Lipid-lowering treatment	0.63 (0.40–0.99)	**0**.**044**	0.35 (0.13–0.95)	**0**.**039**

Cox regression analysis was performed. Hazard ratios (HRs) with 95% confidence intervals (95%CIs) for the time to the event were showed. The adjusted models were with adjustment for age, sex, BMI, hypertension, diabetes, smoking family history of CAD and hs-CRP. FH, familial hypercholesterolemia; LDL-C, low-density lipoprotein cholesterol; Lp(a), Lipoprotein(a); VHR, very high risk.

## Discussion

The main novel findings included the following: (1) approximately 6.5% of the VYPs with MI were detected as definite/probable FH. The detection rate in group on lipid-lowering medication or patients at age of ≤25 years of the very young infarction patients was up to 12.6% and 10.3%, respectively. (2) The definite/probable FH patients were characterized by similar levels of comorbidity (hypertension, diabetes, obesity, smoking) but they were more likely to be VHR and had higher coronary severity compared to those with unlikely FH. (3) Most of the patients with FH were treated with cholesterol-lowering pharmacotherapy prior to the admission but none of them reached the optimal LDL-C goal. Nevertheless and noteworthy, the lipid-lowering medication, even if in an undertreated condition would benefit the patients from heavy risk burden in patients with definite/probable FH. (4) Within a median follow up of 40 months, the risk of recurrent events in definite/probable FH was not significantly different to that in unlikely FH. While the prior lipid-lowering medication and coronary severity were significantly associated with the outcomes. Together, these findings might underscore the importance or need of early identification and treatment of clinical FH in VYPs with MI, a severe population^27^.

Previously, several studies have reported on this issue. From Greece in 320 consecutive patients who had survived their first STEMI ≤35 years of age, they identified one of five patients with definite/probable HeFH and pointed out a therapeutic gap and a high risk of recurrence events in HeFH patients^28^. From shanghai, a cohort of somewhat older patients (<55 years of age for male and <60 years of age for female) with a sample of 498 STEMI showed a lower prevalence of FH (as expected since patients were older)^29^. Further, it was reported a putative prevalence of 1:5 in patients with premature coronary heart disease (<50 years of age) in both sexes from an analysis of EUROASPIRE IV trial^30^. In the present study, we should point out that the FH defined was not equal to the true FH. A comparison of phenotypic variation in heFH with the same or similar mutations in the LDL receptor gene between Chinese natives or Canadian Chinese was reported as early as 1998^31^. The research showed that the Chinese natives exhibit a milder phenotype, such as lower LDL-C levels, less prevalence of tendon xanthomata or premature CAD, than the Chinese living in Canada. The authors also found that Chinese heFH living in Canada exhibit a phenotype similar to that of other FH patients in Western societies, suggesting environmental factors such as diet with a significant role in modulating the phenotypic variation. Owing to the mild heFH phenotype of Chinese, the data on how well the phenotype FH by Dutch FH criteria perform in Chinese population to catch true FH was unknown. Most importantly, the phenotypic variation might lead to underdiagnosis and undertreatment of FH in Chinese with Dutch criteria. Therefore, the importance of the study was seemed not to just represent the common FH phenotype but to underscore the urgent need to promote appropriate diagnostic screening of FH and to improve early and aggressive clinical management in China of this extremely high-risk condition.

Early recognition of FH is important to both introduce proper medical treatment and perform screening and/or counseling to reduce cardiovascular risk later in life in this population. Our data suggested that the prevalence of FH in patients under age of 25, 26–30, 31–35 years were 10.3%, 6.9% and 6.0% respectively. The FH group were found to be younger than unlikely FH. However, the awareness of the affected patients is very poor, especially in China^26^. Lack of proper training of medical professionals and costs of widespread screening to discover FH cases are also the reason for great concern. To improve the awareness, treatment, and control of FH, both patients and cardiologist, or both hospitals and government can play an important role in providing coverage for routine cholesterol screening and make lipid-lowering medications affordable.

The present study suggested the notion that high levels of cholesterol was generally well recognized among medical providers, but an underdetection and therapeutic shortfall in treatment to recommended LDL-C plasma levels was indicated in our population. supported by the fact that only 60.0% of the definite/probable FH patients received cholesterol-lowering treatment 3 months before baseline and none of them reached the cholesterol goal, despite these patients having a high cardiovascular risk. However, such prior therapy remained critical for managing atherogenic lipids and reducing cardiovascular risk, particularly in the context of medical care for FH patients. This supports that the lipid-lowering medications both low dose used and late time initiated could reduce the risk burden in FH. More benefit from the intensive or early treatment was hence not difficult to speculate^32–34^. New class of lipid-lowering drugs, might be a promising strategy in FH patients not achieving LDL-C targets or not tolerating statins.

Interestingly, our data indicate that advanced MI is more common in young men. In fact, the scenario on gender difference in the present study was not unexpected. The finding confirmed the existence of the so called ‘gender paradox’ in young patients with MI. Several mechanisms might be involved to explain this phenomenon. Firstly, the finding was largely accordance with previous studies, which is possibly associated with the low incidence of CAD in premenopause women. Secondly, young men were more likely to have unhealthy lifestyles and unfavourable psychosocial factors compared with women and these risk factors might accelerate the CAD development. In addition, our study and many other studies on CAD have included mostly men, the knowledge on the associations in women might be in need of further specific evaluation.

A major limitation of the study was the lack of access to mutation testing as previously discussed. The LDL-C levels for FH diagnosis were the estimated values rather than the true untreated LDL-C for the medical-treated patients, and might have certain bias.

## Conclusion

In conclusion, our study as the largest sample at present showed that FH was detected to be relatively frequent among VYPs with the first MI. Most importantly, patients with prior lipid-lowering treatment even in an underused condition would reduce the coronary burden and improve the outcome. Our findings supported intensive effort to the FH screening and advance statin therapy in this severe population.

## Electronic supplementary material

Supplemental Materials
