# Supplementary material for: Familial hypercholesterolemia in very young myocardial infarction
Source: Sci Rep. 2018 Jun 11;8:8861. doi: 10.1038/s41598-018-27248-w (PMC5995844; doi:10.1038/s41598-018-27248-w)
Supplement: Supplementary file 1 — Supplemental Materials [file 41598_2018_27248_MOESM1_ESM.doc]

# Title: Familial hypercholesterolemia in very young myocardial infarction

**Running Title:** FH in young MI

**Authors:** Sha Li,^#^ Hui-Wen Zhang,^#^ Yuan-Lin Guo, ^#^ Na-Qiong Wu, ^#^ Cheng-Gang Zhu, Xi Zhao, Di Sun, Xiong-Yi Gao, Ying Gao, Yan Zhang, Ping Qing, Xiao-Lin Li, Jing Sun, Geng Liu, Qian Dong, Rui-Xia Xu, Chuan-Jue Cui, Jian-Jun Li*

^#^ These authors contribute equally to this study

**Statement of authorship:** All authors above take responsibility for all aspects of the reliability and freedom from bias of the data presented and their discussed interpretation.

***Correspondence:** Professor Jian-Jun Li, MD, PhD. **Tel:** 86+10+88396077; **Fax:** 86+10+88396584; **E-mail:** [lijianjun938@126.com](mailto:lijianjun938@126.com).

**Supplemental Figures**

**Supplemental Figure 1．**Prevalence of FH according to unadjusted LDL-C and Lp(a)-adjusted LDL-C. FH, familial hypercholesterolemia; LDL-C, low-density lipoprotein cholesterol; Lp(a), lipoprotein (a).

**Supplemental Figure 2.** Time-to-event analysis comparing among different FH phenotype. FH, familial hypercholesterolemia.

**Supplemental Figure 1**

**
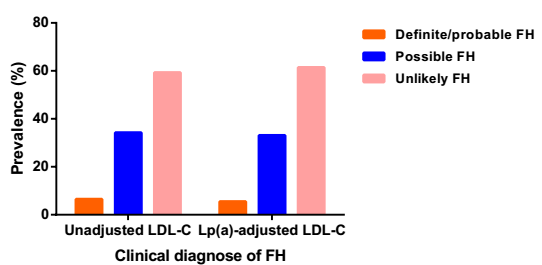
**

**Supplemental Figure 2**

**
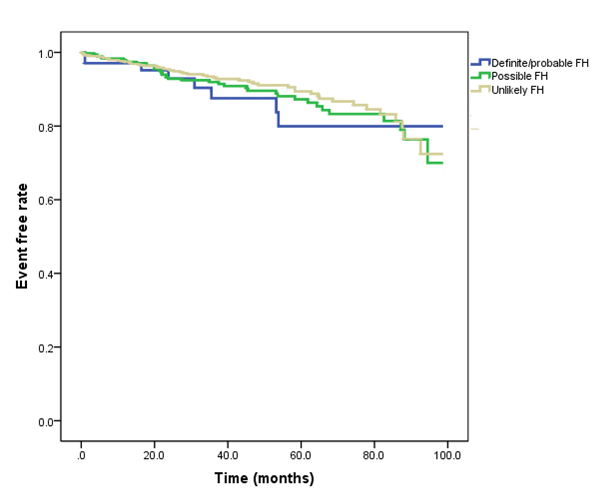
**
